# In vivo evaluation of the effects of combined boron and gadolinium neutron capture therapy in mouse models

**DOI:** 10.1038/s41598-022-17610-4

**Published:** 2022-08-03

**Authors:** Woonghee Lee, Kyung Won Kim, Jeong Eun Lim, Swarbhanu Sarkar, Jung Young Kim, Yongmin Chang, Jeongsoo Yoo

**Affiliations:** 1 Department of Molecular Medicine, Brain Korea 21 Four KNU Convergence Educational Program of Biomedical Sciences for Creative Future Talents, School of Medicine, Kyungpook National University, Daegu, 41944 Republic of Korea; 2 Division of Applied RI, Korea Institute of Radiological and Medical Sciences, Seoul, 01812 Republic of Korea

**Keywords:** Nanoparticles, Cancer therapy, Drug discovery, Medical research

## Abstract

While boron neutron capture therapy (BNCT) depends primarily on the short flight range of the alpha particles emitted by the boron neutron capture reaction, gadolinium neutron capture therapy (GdNCT) mainly relies on gamma rays and Auger electrons released by the gadolinium neutron capture reaction. BNCT and GdNCT can be complementary in tumor therapy. Here, we studied the combined effects of BNCT and GdNCT when boron and gadolinium compounds were co-injected, followed by thermal neutron irradiation, and compared these effects with those of the single therapies. In cytotoxicity studies, some additive effects (32‒43%) were observed when CT26 cells were treated with both boron- and gadolinium-encapsulated PEGylated liposomes (B- and Gd-liposomes) compared to the single treatments. The tumor-suppressive effect was greater when BNCT was followed by GdNCT at an interval of 10 days rather than vice versa. However, tumor suppression with co-injection of B- and Gd-liposomes into tumor-bearing mice followed by neutron beam irradiation was comparable to that observed with Gd-liposome-only treatment but lower than B-liposome-only injection. No additive effect was observed with the combination of BNCT and GdNCT, which could be due to the shielding effect of gadolinium against thermal neutrons because of its overwhelmingly large thermal neutron cross section.

## Introduction

Neutron capture therapy (NCT) is a binary radiation therapy modality based on a nuclear reaction that occurs when stable isotopes are irradiated with neutrons to produce high-energy particles and gamma (γ)-rays^1,2^. NCT includes two methods: boron neutron capture therapy (BNCT) and gadolinium neutron capture therapy (GdNCT). BNCT is based on the nuclear reaction between boron-10 (^10^B) and thermal neutrons (^10^B[n,α]^7^Li), resulting in high-linear-energy-transfer (LET) alpha (α) particles (^4^He) and recoiling ^7^Li nuclei with an average total kinetic energy of 2.33 MeV^3,4^. ^10^B (natural abundance 19.9%) has a relatively high thermal neutron cross section (3800 barns). Because α particles have very short path lengths (5–9 μm), their destructive effect is limited to boron-containing cells^1,2^. Thus, theoretically, α particles can selectively destroy tumor cells and spare adjacent normal cells.

In contrast, GdNCT is primarily based on a (^157^Gd[n,γ]^158^Gd) nuclear reaction, resulting in the emission of long-range, prompt γ-rays, internal conversion electrons, characteristic X-rays, and Auger electrons with high total kinetic energy (7.94 MeV)^3^. ^157^Gd (natural abundance 15.7%) has an extremely large thermal neutron cross section (255,000 barns), which is 66 times higher than that of ^10^B^5–7^. While BNCT depends primarily on the short flight range of α particles emitted by the boron neutron capture reaction, GdNCT mainly relies on long-range gamma rays released by the gadolinium neutron capture reaction. Even if Gd is extracellularly present in tumor tissues, it has an extensive effect on tumors^3^. Moreover, intensive destruction of DNA in tumor cells can be achieved by short-range and high-LET Auger electrons emitted by the ^157^Gd neutron capture reaction when the gadolinium agent is within the nuclei of tumor cells^8^.

These complementary, tumor-killing mechanisms of BNCT and GdNCT raise the question of whether the combination of BNCT and GdNCT could increase therapeutic efficacy and reduce side effects towards normal tissue^9^. However, only few combinations of BNCT and GdNCT therapies have been reported^3,9–11^ and in vivo studies of such combined therapies are rare^12,13^.

Here, we report an in vivo evaluation of the tumor-suppressive effect in a tumor-bearing mouse model when boron and gadolinium compounds were co-injected, followed by thermal neutron irradiation, and a comparison of their therapeutic effects with those of BNCT and GdNCT administered separately.

## Materials and methods

### Materials

**Figure 1:**
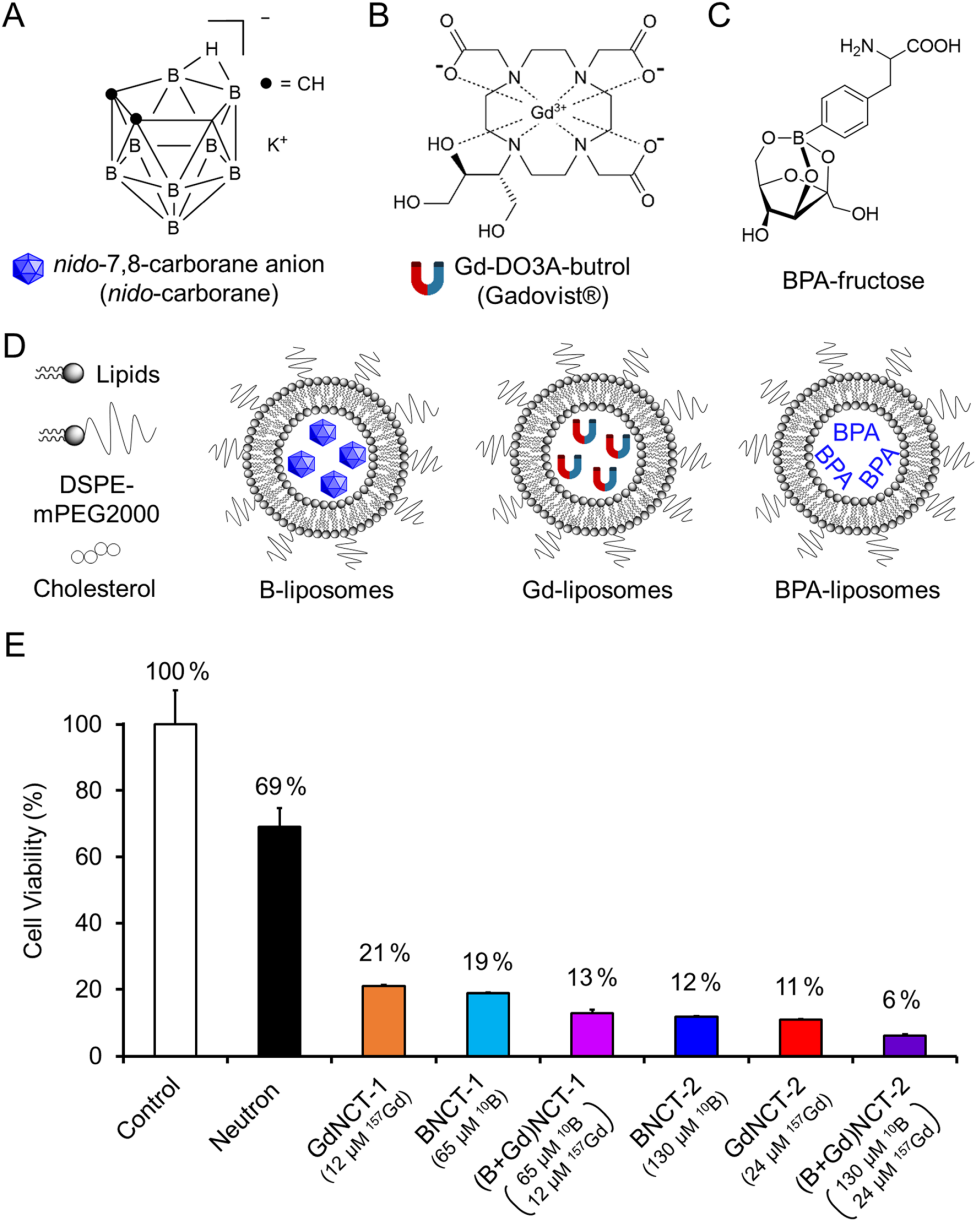
Schematic profiles of liposomes used in neutron capture therapy and in vitro cytotoxicity tests. Chemical structures of (**A**) *nido*-7,8-carborane potassium salt (*nido*-carborane), (**B**) Gd-DO3A-butrol (Gadovist), and (**C**) 4-dihydroxyborylphenylalanine‒fructose complex (BPA‒fructose). (**D**) Schematic illustration of *nido*-carborane-encapsulated liposomes (B-liposomes), Gadovist-encapsulated liposomes (Gd-liposomes), and BPA‒F-encapsulated liposomes (BPA-liposomes). (**E**) Cytotoxicity of B- and Gd-liposomes in CT26 cells upon neutron irradiation (n = 3).

*Ortho*-carborane was purchased from Katchem (Prague, Czech Republic) and potassium *nido*-7,8-carborane (K[7,8-C_2_B_9_H_12_]), hereafter referred to as *nido*-carborane (Fig. 1A), was prepared according to a previously reported method^14^. Gadovist® (Gd-DO3A-butrol, Fig. 1B) was purchased from Bayer Pharmaceuticals (Berlin, Germany). All lipids were purchased from Avanti Polar Lipids, Inc. (Alabaster, AL, USA). RPMI 1640 medium, phosphate-buffered saline (PBS), and fetal bovine serum (FBS) were purchased from HyClone (Logan, Utah, USA). All other chemicals, solvents, and reagents were purchased from Sigma-Aldrich (St. Louis, MO, USA). Murine colon adenocarcinoma 26 (CT26) cells were obtained from the American Type Culture Collection (cat. # CRL-2638, ATCC, VA, USA). The thermal-neutron beam was generated using an MC-50 cyclotron (Scanditronix, Sweden) by irradiating a beryllium target with a proton beam (20 μA, 35 MeV) at the KIRAMS in Seoul, Korea. The generated thermal-neutron flux was approximately 1.94 × 10^4^ neutrons/cm^2^·s^15,16^. B-liposomes containing *nido*-carborane (BNCT-1,2) and Gd-liposomes containing Gadovist (GdNCT-1,2) were prepared, according to the methods described in previous reports^14,17^.

### Preparation of liposomes with encapsulated BPA‒fructose

First, 4-dihydroxyborylphenylalanine‒fructose complex (BPA–F) was prepared according to the literature (Fig. 1C)^18^. Briefly, BPA (120 mg, 0.61 mmol) was mixed with MilliQ water (2 mL) in a round bottom flask at room temperature. The pH of the reaction medium was then adjusted to ~ 10 with aqueous NaOH (10 M). The solution was then stirred for 10 min, followed by the addition of fructose (114 mg, 0.63 mmol). The resulting mixture was then stirred at room temperature for 20 min and neutralized to pH 7.4 by the careful addition of concentrated HCl (***caution***: special care was taken to maintain a low temperature during the addition of HCl by placing the reaction vessel in ice-cold water). The solution was then allowed to stand at room temperature for 16 h, and the total volume was finally adjusted to 5 mL with MilliQ water. The solution was filtered through a 0.22 µm syringe filter to obtain a clear solution of BPA–F. This solution was directly used to hydrate thin lipid films during liposome preparation. DPPC (5 mg, 6.8 μmol), DPPG (5 mg, 6.7 μmol), cholesterol (2 mg, 5.0 μmol), and DSPE-mPEG2000 (9 mg, 3.4 μmol) were dissolved in chloroform. A thin lipid film was formed on the inner surface of a round bottom flask by chloroform evaporation and hydrated with the prepared BPA–F solution (5 mL) at 50 °C. The resulting mixture was frozen and thawed for five cycles. BPA‒F-encapsulated PEGylated liposomes (BPA-liposomes) were prepared by extrusion using a series of polycarbonate membrane filters (Whatman, Kent, UK) and a mini extruder (Avanti Polar lipids, AL, USA). Unencapsulated BPA‒F was removed using an Ultracel centrifugal filter (molecular weight cut-off 100, Millipore, MA, USA). The purified BPA‒liposomes were suspended in PBS. In addition, *nido*-carborane-encapsulated PEGylated liposomes (B-liposomes) and Gadovist-encapsulated PEGylated liposomes (Gd-liposomes) were prepared using a thin-film-and-hydration method according to a previously reported procedure (Fig. 1D)^14,17^.

### Cell culture and cytotoxicity tests

CT26 cells were cultured in RPMI 1640 medium supplemented with 10% FBS and 1% antibiotics at 37 °C in a humid atmosphere containing 5% CO_2_. The cells were seeded in T25 culture flasks (1 × 10^5^ cells per flask) and cultured overnight. Then, each flask was treated with B-liposomes (65 µM or 130 µM ^10^B) or Gd-liposomes (12 µM or 24 µM ^157^Gd). In addition, combinations of B- and Gd-liposomes (65 µM ^10^B + 12 µM ^157^Gd or 130 µM ^10^B + 24 µM ^157^Gd) were added to each flask to evaluate the effects of combined BNCT and GdNCT at the cellular level. The flasks were washed with PBS to remove noninternalized B- and Gd-liposomes after 1 h of incubation and irradiated with thermal neutrons for 12 min. The cytotoxicity was evaluated using the 3-(4,5-dimethylthiazol-2-yl)-2,5-diphenyltetrazolium bromide) tetrazolium (MTT) assay. MTT (5 mg/mL) in PBS was added to each flask, followed by incubation for 2 h. The absorbance of the formazan crystals formed in the flasks was measured using a spectrophotometer (SPECTROstar Nano, BMG Labtech, Ortenberg, Germany) at 550 nm. The viability of the CT26 cells with and without thermal-neutron irradiation but without B- or Gd-liposome treatment was also evaluated as a control.

### Quantification of intracellular boron and gadolinium concentration in CT26 cells

CT26 cells were seeded into 6-well plates and incubated overnight. The cells were then exposed to B-liposomes (65 μM or 130 μM ^10^B; BNCT-1 and BNCT-2, respectively), Gd-liposomes (12 μM or 24 μM ^157^Gd; GdNCT-1 and GdNCT-2, respectively), or combined B- and Gd-liposomes (65 μM ^10^B + 12 μM ^157^Gd or 130 μM ^10^B + 24 μM ^157^Gd; (B + Gd)NCT-1 and (B + Gd)NCT-2, respectively). After 1 h of incubation, the cells were washed twice with PBS, trypsinized, and centrifuged at 200 × *g* for 3 min. The cell pellets were added to nitric acid and hydrogen peroxide at final concentrations of 40% and 5% w/v, respectively, followed by heating at 90 °C for 1 h to induce complete digestion. The level of boron and gadolinium uptake by the cells was determined using inductively coupled plasma atomic emission spectroscopy (ICP-AES; Optima 7300DV, Perkin Elmer, USA).

### Animal tumor xenograft models

All animal procedures were performed in accordance with appropriate standards under a protocol approved by the Animal Ethics Committee requirements of Kyungpook National University (approval no. 2017-0096). The reporting in this manuscript follows the ARRIVE guidelines. To prepare CT26 tumor models, 5 × 10^6^ CT26 cells were subcutaneously injected into the left thigh of five-week-old female BALB/c mice (16–19 g, Hyochang Bioscience, Daegu, Korea). When the tumor volumes reached 50–100 mm^3^, the CT26 tumor-bearing mice were subjected to combined BNCT and GdNCT treatments.

### Quantification of tissue uptake concentration of boron and gadolinium in animal models

CT26 tumor-bearing mice were intravenously injected with B-liposomes (21 mg ^10^B per kg for BNCT-2) or Gd-liposomes (38 mg ^157^Gd per kg for GdNCT-2). The combined boron and gadolinium groups were simultaneously injected with B- and Gd-liposomes (21 mg ^10^B + 38 mg ^157^Gd per kg for (B + Gd)NCT-2). One hour post-injection, the mice were sacrificed, and the tumor and muscle were harvested. The wet weight of each sample was measured immediately after collection. These samples were then added to nitric acid and hydrogen peroxide at final concentrations of 40% and 5% w/v, respectively, followed by heating at 90 °C for 3 h to induce complete digestion. The level of boron and gadolinium uptake in the tissue was determined using ICP-AES, as above.

### In vivo evaluation of the therapeutic effect of the combination of BNCT and GdNCT

Before neutron-beam irradiation, the CT26 tumor-bearing mice were anesthetized with a combination of ketamine (100 mg/kg) and xylazine (20 mg/kg) by intraperitoneal injection. A 12-mm-thick polyethylene board was prepared to protect the major organs of the mice, except the left thigh bearing the CT26 tumor, from thermal-neutron irradiation. Each tumor model was irradiated with thermal neutrons at a flux of 1.94 × 10^4^ neutrons/cm^2^·s for 20 min. Tumor volumes were measured using calipers and calculated as (A × B^2^)/2, where A and B are the longest and shortest dimensions of the tumor (mm), respectively.

First, to evaluate the combined therapeutic effect of BNCT and GdNCT, two separate injections of B-liposomes or Gd-liposomes were performed at an interval of 10 days. Tumor-bearing mice were randomly divided into six groups (n = 4): control, neutron-only (neutron), BNCT-22, GdNCT-22, Gd-BNCT, and B-GdNCT. The tail veins of the mice in the BNCT-22 or GdNCT-22 groups were injected twice with B-liposomes (21 mg ^10^B per kg) or Gd-liposomes (38 mg ^157^Gd per kg), respectively, at an interval of 10 days. Gd-BNCT groups were intravenously injected with Gd-liposomes (38 mg ^157^Gd per kg) at the time of the first neutron irradiation and then with B-liposomes (21 mg ^10^B per kg) at the time of the second neutron irradiation. Conversely, B-GdNCT groups were injected with B-liposomes (21 mg ^10^B per kg) at the time of the first neutron irradiation and then with Gd-liposomes (38 mg ^157^Gd per kg) at the time of the second neutron irradiation. Tumor volumes and body weights were measured every other day after the first neutron irradiation until the 31st day. These values were expressed as mean ± SD (n = 4). The weights of the excised tumor tissues from euthanized tumor-bearing mice were measured to compare the therapeutic effects.

Next, B-liposomes and Gd-liposomes were simultaneously injected to determine whether the combination had any synergistic effect compared to the BNCT-only and GdNCT-only groups. Tumor-bearing mice were randomly divided into eight groups (n = 5): control, neutron, BNCT-1, BNCT-2, GdNCT-1, GdNCT-2, (B + Gd)NCT-1, and (B + Gd)NCT-2. The tail veins of the mice in the BNCT groups were injected with B-liposomes (11 mg ^10^B per kg for BNCT-1 and 21 mg ^10^B per kg for BNCT-2). The GdNCT groups were intravenously injected with Gd-liposomes (19 mg ^157^Gd per kg for GdNCT-1 and 38 mg ^157^Gd per kg for GdNCT-2). The combined boron and gadolinium (B + Gd)NCT groups were simultaneously injected with B- and Gd-liposomes (11 mg ^10^B + 19 mg ^157^Gd per kg for (B + Gd)NCT-1 and 21 mg ^10^B + 38 mg ^157^Gd per kg for (B + Gd)NCT-2). Tumor volumes, body weights, and survival were measured every other day until day 35 after thermal-neutron beam irradiation. Then, the weights of the excised tumor tissues from the euthanized tumor-bearing mice were measured.

Finally, we used different boronylated liposomes in the combined boron and gadolinium neutron capture therapy. Instead of *nido*-carborane, BPA‒F-encapsulated PEGylated liposomes (BPA-liposomes) were used in BNCT studies. For these BNCT studies, tumor-bearing mice were randomly divided into eight groups (n = 5): control, neutron, BNCT-3, BNCT-4, GdNCT-1, GdNCT-2, (B + Gd)NCT-3, and (B + Gd)NCT-4. Two different concentrations of BPA-liposomes were intravenously injected into the BNCT groups, while BPA-liposomes and Gd-liposomes were simultaneously injected into the (B + Gd)NCT groups. The tumor volumes and body weights were measured every other day until day 21 after thermal-neutron beam irradiation. Then, the weights of the excised tumor tissues from the euthanized tumor-bearing mice were measured.

## Results and discussion

### Cytotoxicity of B-, Gd-, (B + Gd)-liposomes upon neutron irradiation

First, we evaluated the in vitro effect of combined BNCT and GdNCT at the cellular level. For this purpose, murine CT26 colon cancer cells were treated with two different concentrations of *nido*-carborane-encapsulated PEGylated liposomes (B-liposomes) and/or Gadovist-encapsulated PEGylated liposomes (Gd-liposomes) along with neutron irradiation (Fig. 1E). When CT26 cells were simultaneously treated with low-dose B- (65 µM ^10^B) and Gd-liposomes (12 µM ^157^Gd), their viability was reduced to only 12.8 ± 1.2% of the control group, which was 32% and 39% lower than the viability observed with single treatments with B- and Gd-liposomes, respectively. When CT26 cells were simultaneously treated with high-dose B- and Gd-liposomes (130 µM ^10^B, 24 µM ^157^Gd), viability was 47% and 43% lower than that observed with single BNCT and GdNCT treatments. The increased therapeutic efficacy of the combined BNCT and GdNCT at the cellular level might be attributed to the α particles and high-LET Auger electrons emitted by the neutron capture reaction of ^10^B and ^157^Gd^9^.

The intracellular concentrations of boron and gadolinium in CT26 cells were directly measured using ICP-AES (Table 1). The boron (^10^B) concentrations were 7.1 ± 1.6, 73.7 ± 4.9, 37.9 ± 1.3, and 73.5 ± 1.1 ppm for low-dose B-liposomes (BNCT-1), high-dose B-liposomes (BNCT-2), low-dose combined B- and Gd-liposomes ((B + Gd)NCT-1), and high-dose combined B- and Gd-liposomes ((B + Gd)NCT-2), respectively. The gadolinium (^157^Gd) concentrations were 21.9 ± 3.7, 35.9 ± 4.4, 17.0 ± 1.4, and 32.8 ± 0.9 ppm for low-dose Gd-liposomes (GdNCT-1), high-dose Gd-liposomes (GdNCT-2), low-dose combined B- and Gd-liposomes ((B + Gd)NCT-1), and high-dose combined B- and Gd-liposomes ((B + Gd)NCT-2), respectively. The intracellular concentrations following simultaneous treatment with B- and Gd-liposomes were comparable to those following single treatment with B- or Gd-liposomes. This indicates that B- and Gd-liposomes can be internalized simultaneously into CT26 cells without interfering with each other. Treatment with higher doses of B- and Gd-liposomes also increased the intracellular concentrations of boron and gadolinium, respectively.

**Table 1:** Intracellular boron and gadolinium concentrations (ppm) in CT26 cells determined using inductively coupled plasma atomic emission spectroscopy.

	BNCT-1	BNCT-2	GdNCT-1	GdNCT-2	(B + Gd)NCT-1	(B + Gd)NCT-2
^10^B	7.1 ± 1.6	73.7 ± 4.9			37.9 ± 1.3	73.5 ± 1.1
^157^Gd			21.9 ± 3.7	35.9 ± 4.4	17.0 ± 1.4	32.8 ± 0.9

Data are presented as mean ± standard deviation (SD) of triplicates.

### In vivo evaluation of the therapeutic effect of combined BNCT and GdNCT

**Figure 2:**
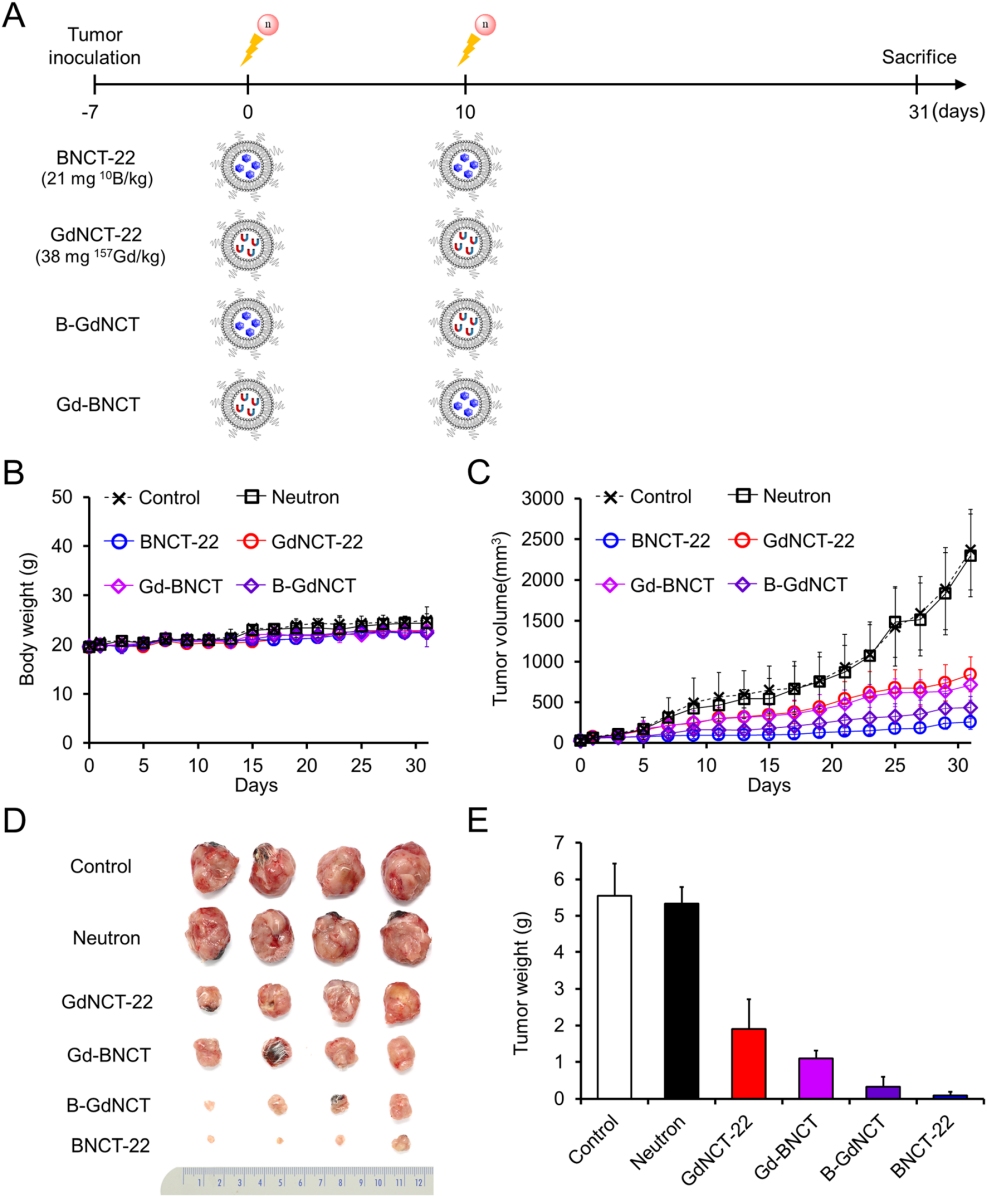
Results of single and combined BNCT and GdNCT using B-liposomes and Gd-liposomes in CT26 tumor-bearing mice (n = 4). (**A**) General experimental procedure for double injection and irradiation. (**B**) Body weights of each group after first neutron irradiation. (**C**) Tumor growth curves of each group after first neutron irradiation. (**D**) Photographs of tumors resected 31 days after the first neutron irradiation. (**E**) Average weights of tumors resected on day 31.

First, we compared the therapeutic effects of two separate injections of boron or gadolinium at an interval of 10 days (Fig. 2A). The body weight and tumor size of 8 groups of tumor-bearing mice (n = 4 each) were monitored for 31 days after the first neutron irradiation. There was no significant difference in the body weights of any group for 10 days after the first irradiation. Compared to the six neutron therapy groups, the body weights of the control and neutron-only groups were ~ 10% higher after the second neutron irradiation (Fig. 2B). There was no difference in body weight between the boron and gadolinium neutron therapy groups. However, significant differences in tumor suppression were observed in the neutron capture therapy groups depending on the materials injected (Fig. 2C,D). Although a higher amount of gadolinium (38 mg ^157^Gd per kg, GdNCT-22) was injected compared to boron (21.0 mg ^10^B per kg, BNCT-22), tumor suppression was the highest when B-liposomes were injected twice and lowest when Gd-liposomes were injected twice. When boron (B-liposomes) and gadolinium (Gd-liposomes) were injected in sequence (B-GdNCT) or vice versa (Gd-BNCT), the therapeutic effect was higher than that of gadolinium (GdNCT-22) but lower than that of boron (BNCT-22). Interestingly, greater therapeutic efficacy was observed when boron-containing liposomes were injected before gadolinium-containing liposomes than when the order was reversed (Fig. 2E). When the tumors were excised and weighed, the average tumor weight of the B-GdNCT group was only 30% of that of the Gd-BNCT group (335 ± 257 mg vs. 1096 ± 214 mg). This is probably because the therapeutic efficacy of BNCT is higher when the tumor size is small^19^. However, it was difficult to verify the synergistic effect of the combination of BNCT and GdNCT in this study because the therapeutic efficacy of BNCT was much higher than that of GdNCT.

**Figure 3:**
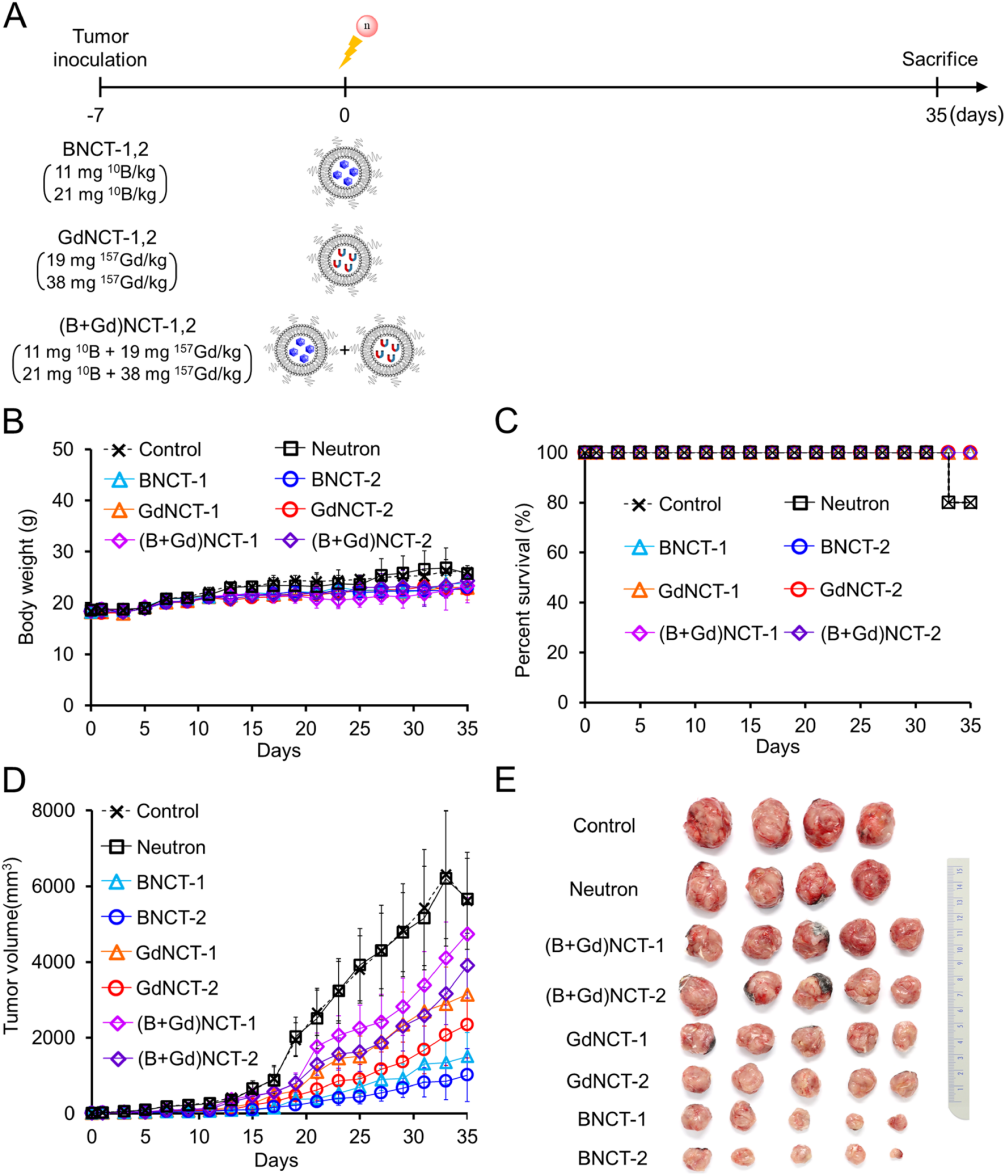
Results of concurrent BNCT and GdNCT combination using B-liposomes and Gd-liposomes in CT26 tumor-bearing mice (n = 5). (**A**) General experimental procedure for injection and irradiation. (**B**) Body weights of each group after neutron irradiation. (**C**) Survival curve of each group up to 35 days after neutron irradiation. (**D**) Tumor growth curves of each group after neutron irradiation. (**E**) Photographs of tumors resected 35 days after neutron irradiation.

In the second experimental setup, B-liposomes and Gd-liposomes were simultaneously injected and their combined effect was examined by comparing tumor sizes with those of BNCT-only and GdNCT-only groups (Fig. 3A). Although more neutron-capturing compounds (boron and gadolinium) were injected in the (B + Gd)NCT groups, the average body weights were comparable with those of other NCT groups (Fig. 3B), indicating no extra toxicity because of the double-injection of B- and Gd-liposomes. When tumor size was continuously monitored every other day, one in five mice died in each of the control and neutron-only groups (Fig. 3C). All mice were sacrificed, and tumors were excised at 35 days post-irradiation. Significant tumor-suppressive effects were seen in NCT groups compared to control and neutron-only groups (Fig. 3D). The highest therapeutic efficacy was observed in the high-dose BNCT group (BNCT-2), followed by the low-dose BNCT group (BNCT-1), and then the high-dose GdNCT (GdNCT-2) and low-dose GdNCT (GdNCT-1) groups (Fig. 3E). To our surprise, the boron and gadolinium combination groups ((B + Gd)NCT-1,2) showed the lowest tumor-suppressive effects. At 35 days post-irradiation, the average tumor volumes of (B + Gd)NCT-1 and -2 groups were 1.5 and 1.7 times larger than those of the GdNCT-1 and -2 groups and 3.1 and 3.8 times larger than those of the BNCT-1 and -2 groups, respectively.

The boron and gadolinium concentrations in tumors and muscles were also quantified directly using ICP-AES (Table 2). As seen in the cell uptake experiments (Table 1), similar uptakes of boron and gadolinium were found in tumors following combination treatment with B- and Gd-liposomes compared with that following the single treatments, demonstrating no interference in each uptake. The boron (^10^B) contents in the tumors of the high-dose BNCT group (BNCT-2) and the B and Gd combination group ((B + Gd)NCT-2) were 152.2 ± 12.1 and 159.5 ± 12.9 µg g^-1^, respectively; this constitutes approximately seven times more than the minimum amount of boron required for a successful BNCT (20 μg of ^10^B per gram of tumor)^1^, demonstrating excellent boron delivery by our liposomal formulation. The gadolinium (^157^Gd) content of the tumor was similar to that of boron: 81.9 ± 6.0 and 101.3 ± 27.1 µg g^-1^ for the high-dose GdNCT (GdNCT-2) and the B and Gd combination groups ((B + Gd)NCT-2), respectively. This also meets the prerequisite gadolinium content for successful GdNCT treatment (50–200 μg ^157^Gd per gram of tumor)^20^. The ICP-AES analysis clearly shows that the relatively lower therapeutic effect of combined boron and gadolinium treatment ((B + Gd)NCT-2) compared with that of the single treatment (BNCT-2 or GdNCT-2) is not due to the lower tumor accumulation of NCT agents. The boron and gadolinium concentrations in muscles were also analyzed as controls (Table 2). The ratio of boron uptake between tumor and muscle was 9.2 and 8.4 for the BNCT-2 and (B + Gd)NCT-2 groups, respectively, which is much higher than the typically acceptable tumor-to-normal tissue ratio, 3^1^. The Gd uptake ratio between tumor and muscle was even higher than that of B (13.2 and 13.0 for GdNCT-2 and (B + Gd)NCT-2, respectively).

**Table 2:** Biodistribution of boron and gadolinium concentrations (µg g^-1^ tissue) in the tumor and muscles of CT26 tumor bearing mice 1 h post-injection determined using inductively coupled plasma atomic emission spectroscopy.

	BNCT-2	GdNCT-2	(B + Gd)NCT-2
**Tumor**			
^10^B	152.2 ± 12.1		159.5 ± 12.9
^157^Gd		81.9 ± 6.0	101.3 ± 27.1
**Muscle**			
^10^B	16.6 ± 7.8		18.9 ± 3.4
^157^Gd		6.2 ± 1.8	7.8 ± 2.7

Data are presented as mean ± standard deviation (SD) of five replicates.

**Figure 4:**
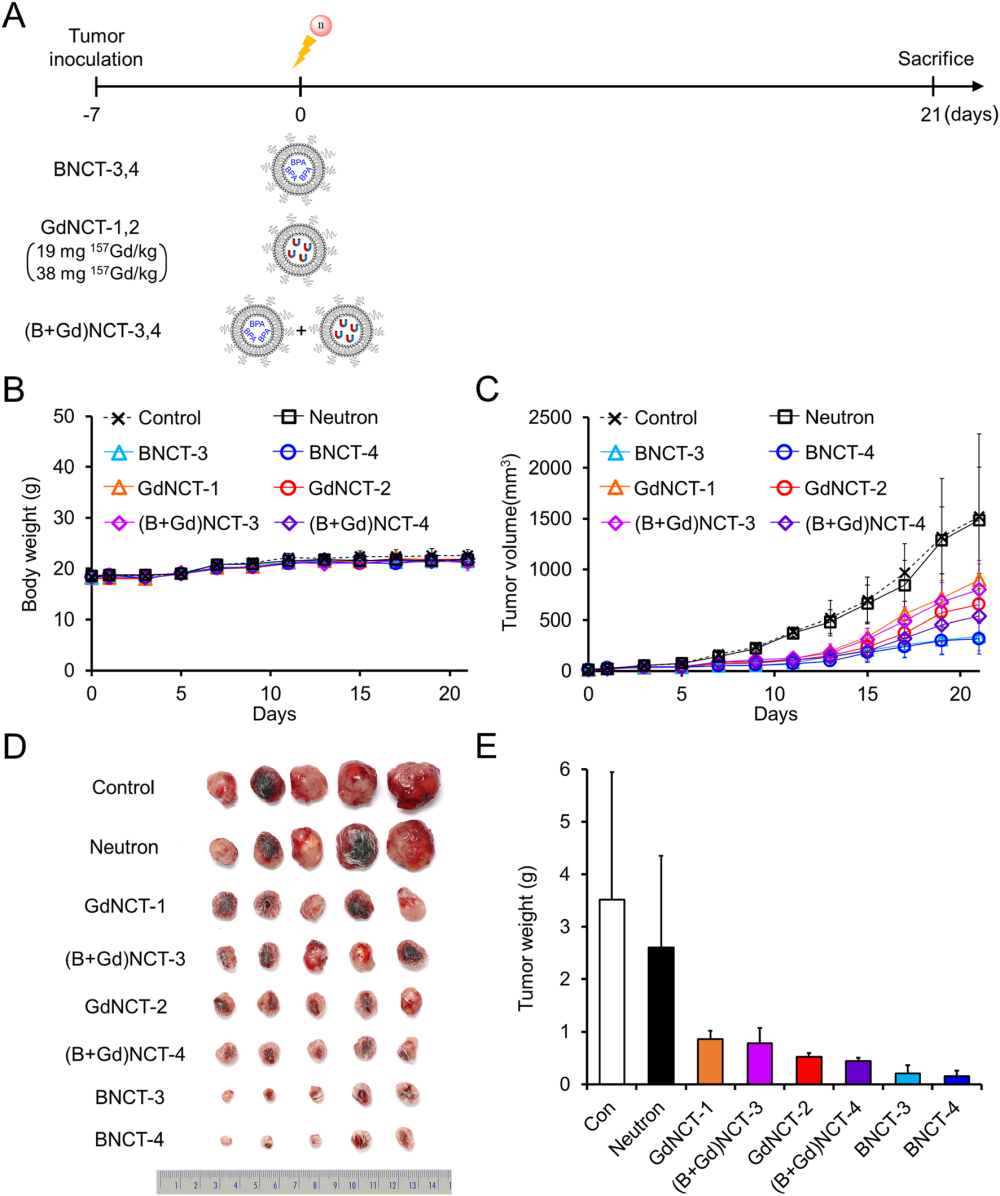
Results of concurrent BNCT and GdNCT combination using BPA-liposomes and Gd-liposomes in CT26 tumor-bearing mice (n = 5). (**A**) General experimental procedure for injection and irradiation. (**B**) Body weights of each group after neutron irradiation. (**C**) Tumor growth curves of each group after neutron irradiation. (**D**) Photographs of tumors resected 21 days after neutron irradiation. (**E**) Average weights of tumors resected on day 21.

Next, we tested another combined boron and gadolinium neutron capture therapy. In this study, we used different boronylated liposomes. Instead of *nido*-carborane, clinically used fructose-conjugated 4-dihydroxyborylphenylalanine (BPA‒F, Fig. 1C) was incorporated into liposomes (BPA-liposomes, Fig. 1D) for BNCT studies (BNCT-3,4, Fig. 4A). The toxicity of the combined BPA and Gadovist neutron therapy was comparable with that of single use of BPA or Gadovist. There was no noticeable difference in body weight between any of the groups for up to 21 days post-irradiation (Fig. 4B). All NCT groups showed significant tumor-suppressive effects (Fig. 4C). Tumor sizes of the control and neutron groups were up to 4.7-fold and at least 1.7-fold greater than those of the high-dose BNCT-4 and low-dose GdNCT-1 groups, respectively (Fig. 4D). The BNCT groups showed the greatest tumor-suppressive effects. When BPA-liposomes and Gd-liposomes were simultaneously injected, the therapeutic efficacy was comparable with that of GdNCT-only groups (Fig. 4E). The average tumor weights of the (B + Gd)NCT-3 and -4 groups were 89% (801 ± 288 mg vs. 898 ± 156 mg) and 83% (539 ± 182 mg vs. 653 ± 154 mg) of those of the GdNCT-1 and -2 groups, respectively.

From the above two studies, it is obvious that when boron compounds were injected simultaneously with gadolinium compounds before neutron irradiation, tumor growth inhibition was lower than when boron compounds were used alone. The therapeutic efficacy of the combined boron and gadolinium neutron capture therapy was comparable to that of gadolinium-only. No synergistic effects were observed with combined BNCT and GdNCT. In conclusion, when boron and gadolinium were co-injected, the therapeutic efficacy of the boron compound disappeared and only the therapeutic efficacy of gadolinium was observed. This could be attributed to the overwhelmingly larger thermal neutron capture cross-section of gadolinium than boron. Gadolinium-157 nuclide has the largest thermal neutron capture cross-section (255,000 b), which is more than 66 times that of B-10^6,7^. More thermal neutrons are captured by gadolinium compounds before any B-10 nuclides, leading to nuclear reaction with only ^157^Gd^3,21^. Matsumura et al. also observed the similar no additive effect at the cellular level when V79 cells were co-treated with boron and gadolinium compounds following neutron irradiation, especially at high Gd concentrations^3^.

In summary, contrary to general expectations, no synergistic effect was observed for combined BNCT and GdNCT. In addition, when boron and gadolinium were co-injected before neutron irradiation, the therapeutic efficacy was comparable to that of GdNCT, but lower than that of BNCT alone. Further detailed control experiments and mechanistic studies, including theoretical calculations, are needed.

## Data Availability

All relevant data are included in the manuscript.
